# Dapoxetine, a Selective Serotonin Reuptake Inhibitor, Suppresses Zika Virus Infection In Vitro

**DOI:** 10.3390/molecules28248142

**Published:** 2023-12-17

**Authors:** Bingzhi Zhang, Jianchen Yu, Ge Zhu, Yun Huang, Kexin Zhang, Xuhan Xiao, Wenxuan He, Jie Yuan, Xiaoxia Gao

**Affiliations:** 1 School of Pharmacy, Guangdong Pharmaceutical University, Guangzhou 510006, China; bingzhi0908@163.com; 2 Key Laboratory of Tropical Disease Control (Sun Yat-sen University), Ministry of Education, Guangzhou 510080, China; yujchen@mail2.sysu.edu.cn (J.Y.); zhug6@mail2.sysu.edu.cn (G.Z.); xiaoxh26@mail2.sysu.edu.cn (X.X.); 3 Department of Biochemistry, Zhongshan School of Medicine, Sun Yat-sen University, Guangzhou 510080, China; 4 School of Basic Medical Sciences, Southern Medical University, Guangzhou 510515, China; wyhl2014@163.com; 5 School of Public Health, Sun Yat-sen University, Guangzhou 510080, China; kxz1106@126.com; 6 School of Life Sciences and Biopharmaceutics, Guangdong Pharmaceutical University, Guangzhou 510006, China; 13078435107@139.com

**Keywords:** Zika virus, dapoxetine, RdRp inhibitor, antiviral

## Abstract

Zika virus (ZIKV) belongs to the *Flavivirus* genus of the *Flaviviridae* family, and is a pathogen posing a significant threat to human health. Currently, there is a lack of internationally approved antiviral drugs for the treatment of ZIKV infection, and symptomatic management remains the primary clinical approach. Consequently, the exploration of safe and effective anti-ZIKV drugs has emerged as a paramount imperative in ZIKV control efforts. In this study, we performed a screening of a compound library consisting of 1789 FDA-approved drugs to identify potential agents with anti-ZIKV activity. We have identified dapoxetine, an orally administered selective serotonin reuptake inhibitor (SSRI) commonly employed for the clinical management of premature ejaculation (PE), as a potential inhibitor of ZIKV RNA-dependent RNA polymerase (RdRp). Consequently, we conducted surface plasmon resonance (SPR) analysis to validate the specific binding of dapoxetine to ZIKV RdRp, and further evaluated its inhibitory effect on ZIKV RdRp synthesis using the ZIKV Gluc reporter gene assay. Furthermore, we substantiated the efficacy of dapoxetine in suppressing intracellular replication of ZIKV, thereby demonstrating a concentration-dependent antiviral effect (EC_50_ values ranging from 4.20 μM to 12.6 μM) and negligible cytotoxicity (CC_50_ > 50 μM) across diverse cell lines. Moreover, cell fluorescence staining and Western blotting assays revealed that dapoxetine effectively reduced the expression of ZIKV proteins. Collectively, our findings suggest that dapoxetine exhibits anti-ZIKV effects by inhibiting ZIKV RdRp activity, positioning it as a potential candidate for clinical therapeutic intervention against ZIKV infection.

## 1. Introduction

Zika virus (ZIKV) is classified within the *Flavivirus* genus of the *Flaviviridae* family and is primarily transmitted through mosquito vectors [1]. The ZIKV was initially discovered in 1947 in rhesus monkeys in the Zika jungle of Uganda, and subsequently identified in human populations in Uganda and Tanzania by 1952 [2,3]. Initially, the global recognition of ZIKV was limited due to its sporadic distribution across different regions worldwide. ZIKV is primarily transmitted through mosquito bites, but it can also be transmitted sexually or passed from mother to child [4]. Early symptoms of ZIKV infection typically include fever, rash, joint and muscle pain, headache, and conjunctivitis [5]. Of particular concern is the infection of pregnant women, which can result in congenital Zika syndrome characterized in infants by microcephaly, meningitis, visual impairment, and neurodevelopmental disorders that impact cognitive abilities, physical mobility, and vision. Consequently, this condition ultimately leads to a reduced life expectancy [6].

Currently, there are no specific antiviral drugs for ZIKV; with treatment primarily focused on symptom management and provision of supportive care. Several vaccine candidates, including mRNA vaccines, DNA vaccines, and subunit vaccines, have exhibited promising outcomes in cellular, murine, or non-human primate experiments, but no vaccines have yet been approved for market use [7,8]. There are only two drugs, namely the monoclonal antibodies Tyzivumab and ZIKV-IG, undergoing clinical trials [9]. Tyzivumab, under clinical investigation, demonstrates potent inhibition of ZIKV infection by impeding viral fusion with host cells. ZIKV-IG contains polyclonal antibodies that target distinct antigenic epitopes, thereby inhibiting the infection of ZIKV [10]. Notably, there is currently a lack of small molecule drugs specifically targeting ZIKV in ongoing clinical trials [11].

ZIKV is an enveloped, single-stranded, positive-sense RNA virus with a genome length of approximately 11 kb. Its genome comprises two non-coding regions known as untranslated regions (UTRs) and an open reading frame (ORF). The translation of the ORF produces a polyprotein of over 3000 amino acid residues, which is subsequently cleaved by host and viral proteases into three structural proteins (capsid protein C, premembrane protein prM, and envelope protein E) and seven nonstructural proteins (NS1, NS2A, NS2B, NS3, NS4A, NS4B, and NS5) [12]. The structural proteins play a crucial role in viral particle assembly, virus-cell adsorption, and invasion, while also encompassing the primary antigenic epitopes of the virus. The nonstructural proteins and non-coding regions are primarily responsible for viral genome replication, translation, and regulation of host immune response and metabolism [13]. Among these proteins, NS5 is the largest in terms of molecular mass and comprises the N-terminal methyltransferase (MTase) and the C-terminal RNA-dependent RNA polymerase (RdRp). The RdRp is involved in the replication and transcription process of ZIKV genome RNA [14]. RdRp is a class of proteins found on all RNA viruses and is key to the replication and transcription of the viral genome, making it a promising target for anti-ZIKV drug research [15]. RdRp has also been targeted in the development of antiviral drugs for other RNA viruses, including Hepatitis C virus (HCV), dengue virus (DENV), and coronaviruses [16]. Significant advancements have been made in the development of viral drugs targeting RdRp, including sofosbuvir for HCV and remdesivir, molnupiravir, and azvudine for coronaviruses [17,18,19].

**Figure 1:**
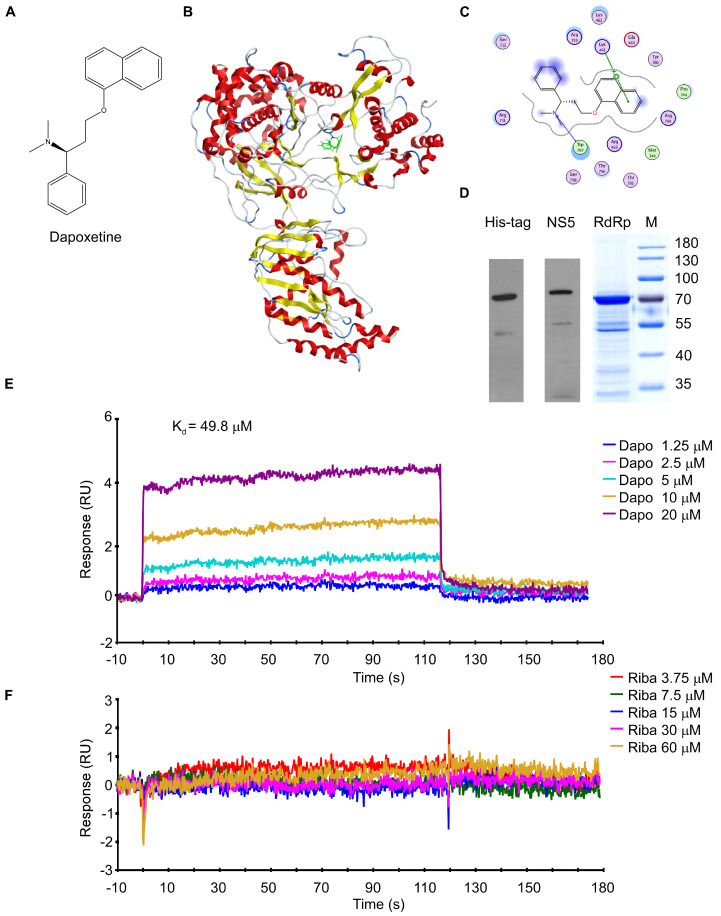
Dapoxetine binds directly to the ZIKV RdRp. (**A**) The chemical structure of dapoxetine. (**B**) An overview of ZIKV NS5 bound to dapoxetine, with dapoxetine shown as green sticks. (**C**) Two−dimensional ligand interaction maps were generated using the Molecular Operating Environment to represent the co−crystals of ZIKV NS5 domains bound with dapoxetine. In the maps, polar residues are colored light purple, charged residues are depicted with an additional blue ring, and lipophilic residues are shown in green. The degree of solvent exposure is indicated by the blue halos. Hydrogen bond interactions with amino acid side chains or the main chain are represented by dashed green arrows, pointing towards the hydrogen bond acceptor. (**D**) Coomassie blue−stained SDS−PAGE (RdRp) of crude extracts and Western blot probed with His−tag and anti−ZIKV NS5 after purification. (**E**,**F**) An SPR assay was conducted to examine and characterize the binding of dapoxetine (**E**) or ribavirin (**F**) to ZIKV RdRp using a BIAcore T100 system. ZIKV RdRp protein was immobilized on a CM5 chip, and serial dilutions of dapoxetine or ribavirin were used as analytes. The dissociation constant (K_d_) values presented in were calculated using the BIAcore T100 analysis software (BIAevaluation Version 3.1).

In this study, we employed a computerized virtual screening model to identify small molecule inhibitors targeting ZIKV RdRp from a compound library consisting of 1789 FDA-approved drugs. We have identified dapoxetine, a selective serotonin reuptake inhibitor (SSRI), as a potential small molecule inhibitor targeting the ZIKV RdRp. Our study has demonstrated that dapoxetine (Figure 1A) effectively inhibits the enzymatic activity of RdRp and exhibits significant antiviral efficacy against ZIKV, suggesting its potential as a promising anti-ZIKV drug clinical application.

## 2. Results

### 2.1. Dapoxetine Was Identified as a ZIKV RdRp Protein Binder through Virtual Screening 

To identify potential compounds with anti-ZIKV activity targeting RdRp, we utilized computational docking methods to simulate compound binding to RdRp and screened a library of 1789 FDA-approved drugs. The crystal structure of ZIKV NS5 protein (PDB: 5TFR) was obtained from the Protein Data Bank and used as the receptor [20], while the active structure of the RdRp domain recognized as the docking site using the site finder in the Molecular Operating Environment (MOE). The collection of compounds was screened for small molecules binding to ZIKV RdRp using the ASE Rescoring function and Triangle Matcher placement method. In the structure-based virtual screen, the top 200 compounds with the highest scores were selected as candidates. Further, we screened the number of ionic bond bindings in the two-dimensional interaction conformation of these compounds bound to the receptor to obtain the higher quality candidates with the best docking results. Among them, dapoxetine was observed to exhibit strong binding with ZIKV RdRp and predicted the formation of 1 H-bond and 1 H-pi-bond between dapoxetine and the RdRp structural domain (Figure 1B,C). The subsequent analysis revealed that dapoxetine specifically binds to the “N” pocket within the priming loop of the ZIKV NS5 protein; this binding occurs at a critical active site in the RdRp structural domain involved in ZIKV NS5-mediated RNA initiation and elongation [21]. The findings suggest that dapoxetine may selectively target specific sites within the ZIKV RdRp pocket, thereby exerting an inhibitory effect on ZIKV RNA replication. In conclusion, our computational docking calculations indicate that dapoxetine exhibits promising potential as a novel small molecule inhibitor targeting ZIKV RdRp to impede viral replication.

### 2.2. Confirmation of Direct Binding between Dapoxetine and ZIKV RdRp

To experimentally validate the direct binding of dapoxetine to ZIKV RdRp, we initially expressed and subsequently purified the RdRp protein from *E. coli* for subsequent investigations. The purified ZIKV RdRp protein was then immobilized on a CM5 chip and subjected to analysis using a BIAcore T100 device (Figure 1D), which utilizes surface plasmon resonance biosensor (SPR) technology to investigate the direct interaction between dapoxetine and RdRp. The SPR data demonstrated direct binding between dapoxetine and the ZIKV protein (K_d_ value = 49.8 μM) (Figure 1E), while the interaction between ribavirin and RdRp exhibited comparatively weaker affinity (Figure 1F). Compared to the K_d_ values of other effective compounds targeting the “N” pocket of ZIKV RdRp (ranges from 10.4 to 49.2 μM) reported previously [22,23,24,25], the results provide compelling evidence that an interaction indeed exists between dapoxetine and ZIKV RdRp.

### 2.3. Dapoxetine Inhibits ZIKV RNA Synthesis

**Figure 2:**
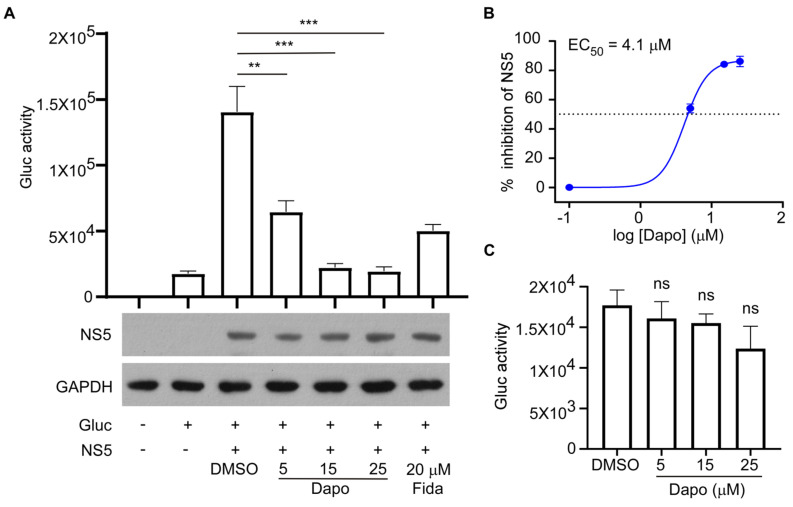
Dapoxetine attenuates ZIKV NS5 RdRp activities. (**A**) The effect of dapoxetine on ZIKV NS5 activity was investigated. HEK293T cells were transfected with a Gluc reporter plasmid or co−transfected with a Gluc reporter and ZIKV NS5 plasmid. The transfected cells were then treated with different concentrations (5, 15, and 25 μM) of dapoxetine for 24 h. Gluc activity in the supernatants was quantified. As a positive control, was used a concentration at 20 μM of fidaxomicin, a potent inhibitor of ZIKV. A negative control was treated with 1% DMSO. Western blot analysis was performed to assess the effect of dapoxetine on the expression level of NS5 (upper panel). GAPDH was used as a loading control (lower panel). (**B**) The EC_50_ of dapoxetine was calculated. (**C**) The effect of dapoxetine on reporter expression was evaluated. HEK293T cells were transfected with a Gluc reporter plasmid and the transfected cells were immediately treated with different concentrations (5, 15, and 25 μM) of dapoxetine or 1% DMSO as a control. Gluc activity was measured 24 h post−transfection. The symbol “+” means inclusion, and “−” means exclusion. Statistical analysis was conducted, and significance levels were denoted as follows: **, *p* < 0.01; ***, *p* < 0.001, “ns” indicates not significant.

To investigate the impact of dapoxetine binding to RdRp on ZIKV RNA synthesis, we utilized the ZIKV Gluc reporter gene system to evaluate the synthesis of ZIKV RNA. In this assay, both the NS5 polymerase of ZIKV and viral RNA containing the Gluc reporter gene were co-expressed in 293 T cells, with the Gluc expression cassette flanked by the 5′ and 3′ untranslated regions (UTRs) of ZIKV. The cellular pol II synthesis initially generated Gluc mRNA, resulting in basal Gluc expression. The amount of Gluc secreted into the medium served as an indicator of viral RNA synthesized by NS5. As a positive control, fidaxomicin (20 μM), a well-established inhibitor of ZIKV RdRp, was included. Our results demonstrated that dapoxetine inhibited Gluc signaling in a dose-dependent manner in the ZIKV Gluc reporter gene assay (Figure 2A) with the EC_50_ being 4.1 μM (Figure 2B). Importantly, dapoxetine did not affect Gluc activity in cells expressing the reporter gene alone (Figure 2C). In summary, we demonstrated that dapoxetine inhibited the activity of ZIKV RdRp and impeded ZIKV RNA generation.

### 2.4. Dapoxetine Inhibits ZIKV Infection In Vitro

**Figure 3:**
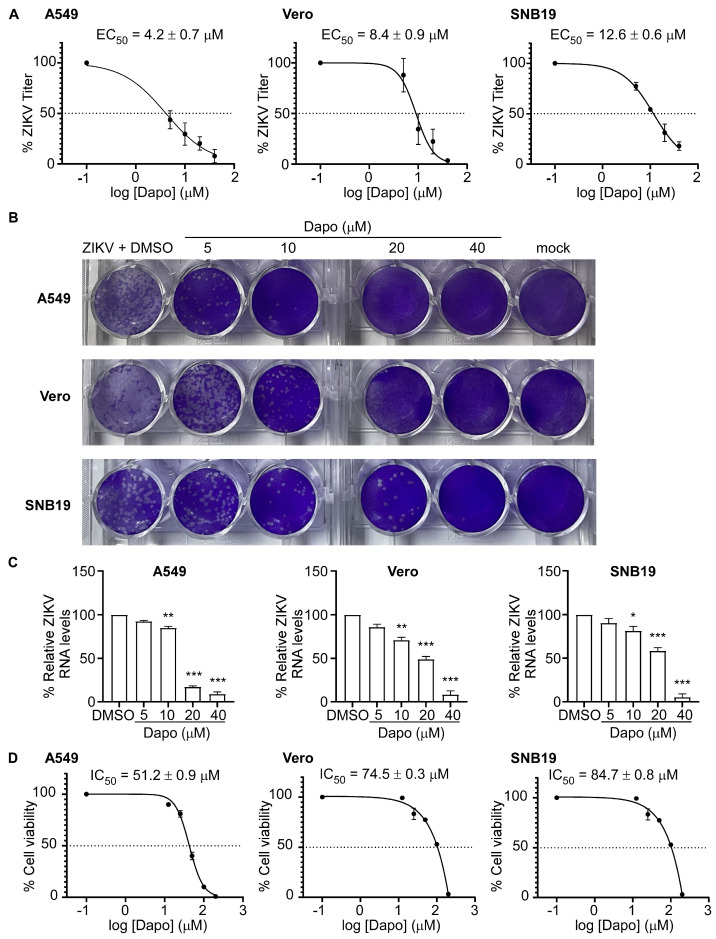
Dapoxetine exhibits inhibitory effects against ZIKV infection in vitro. (**A**) The antiviral spectrum of dapoxetine was evaluated in A549, Vero, and SNB19 cells. ZIKV (ZG−01) titers were quantified using a plaque assay. The data presented are the mean values ± standard deviation from triplicate experiments. (**B**) Representative images of the plaque assay demonstrate the ZIKV (ZG−01) titers in the supernatant, which were detected on new monolayers of Vero cells. The supernatant was obtained from A549, Vero, or SNB19 cell cultures treated with dapoxetine at the indicated concentrations at 48 h post−infection (hpi). (**C**) ZIKV RNA levels were measured using RT−qPCR and normalized to the ZIKV RNA levels in the DMSO control. The presented data are representative of three independent experiments. (**D**) The cytotoxicity spectrum of dapoxetine was evaluated in A549, Vero, and SNB19 cells, while cell viability was assessed using the 3−(4,5−dimethylthiazol−2−yl) −2,5−diphenyltetrazolium bromide (MTT) assay. The data presented are the mean values ± standard deviation from triplicate experiments. Statistical analysis was conducted, and significance levels were denoted as follows: *, *p* < 0.05, **, *p* < 0.01, ***, *p* < 0.001.

In subsequent investigations, we employed plaque reduction assays and RT-qPCR to assess the antiviral efficacy of dapoxetine against ZIKV (ZG-01) infection in various cell lines, including SNB19, A549, and Vero. The results unequivocally demonstrated the potent inhibitory effect of dapoxetine on ZIKV infection, manifesting strong anti-ZIKV activity with the EC_50_ from 4.2 μM to 12.6 μM (Figure 3A,B). Quantification of ZIKV RNA levels was reduced in ZIKV-infected cells following treatment with dapoxetine detected by RT-qPCR, which demonstrated the efficacy of dapoxetine in inhibiting ZIKV RNA replication (Figure 3C). Moreover, the results obtained from the MTT assay indicated that dapoxetine exhibited negligible cellular toxicity (Figure 3D). The above results underscore the promising antiviral potential of dapoxetine against ZIKV, while also highlighting its favorable safety profile. Consequently, further investigation and advancement of dapoxetine as a therapeutic intervention for ZIKV infection is highly warranted in order to broaden the potential treatment alternatives for this viral disease.

### 2.5. Dapoxetine Effectively Reduces ZIKV Infection 

**Figure 4:**
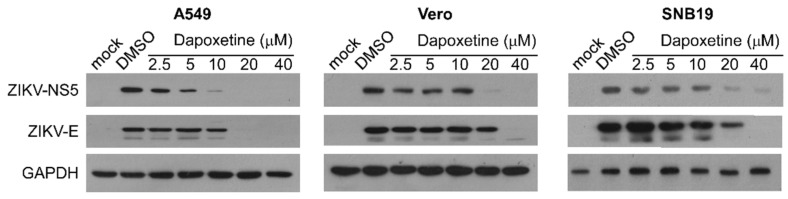
Dapoxetine exhibits inhibitory effects on the expression of ZIKV proteins. Western blotting analysis was performed to assess the protein expression of ZIKV NS5 and E in cell lysates derived from A549, Vero, and SNB19 cells. The cells were treated with dapoxetine or DMSO at indicated concentrations at 48 hpi (MOI = 0.2) to evaluate the anti-ZIKV activity of dapoxetine. The presented data provide insights into the modulation of ZIKV protein expression by dapoxetine. The experiment was independently performed in three replicates, and the one of results is shown above.

To further investigate the antiviral effects of dapoxetine on ZIKV, we conducted immunoblotting analysis to assess the expression of two vital ZIKV proteins in various cell lines. The ZIKV NS5 protein, the largest nonstructural protein in ZIKV, plays a crucial role in viral RNA genome replication. The ZIKV E protein is involved in mediating the fusion of the virus with the host cell membrane. Our results demonstrate a significant and dose-dependent reduction in the expression of both NS5 and E proteins following dapoxetine treatment (Figure 4). The observed decrease in protein expression implies a reduction in the population of cells infected with ZIKV.

**Figure 5:**
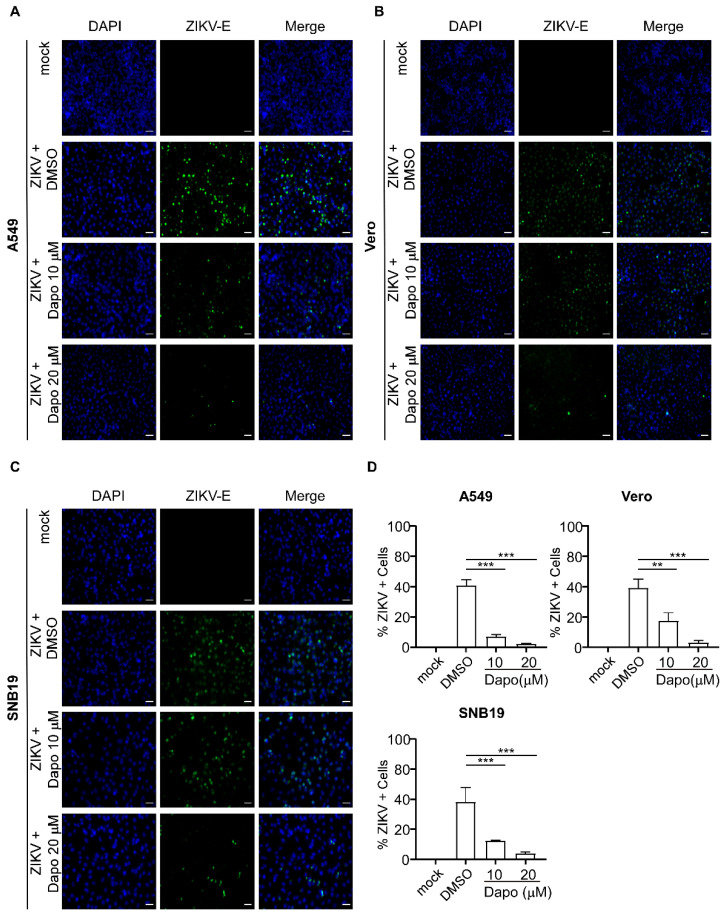
Dapoxetine exhibits preventive effects against ZIKV infection in cells. (**A**–**C**) Immunofluorescence staining for ZIKV-E was performed in A549, Vero, and SNB19 cells (MOI = 2) infected with ZIKV. The infected cells were treated with DMSO or different concentrations (10 and 20 μM) of dapoxetine for 48 h (Scale bar, 50 μm). (**D**) The numbers of ZIKV ^+^ cells relative to mock infection was quantified and presented in the histogram. The counts of total cells (blue) and ZIKV-infected cells (green) in the images were achieved using Image J (1.50i software NIH, Bethesda, MD, USA). Statistical significance levels were indicated: ** *p* < 0.01; *** *p* < 0.001.

To further evaluate the inhibitory effect of dapoxetine on ZIKV infection, we employed immunofluorescence to visualize the antiviral activity of dapoxetine. In the control group, when infected with the virus but treated solely with DMSO, a significant proportion of cells exhibited green fluorescence, indicating a severe ZIKV infection. Conversely, in the dapoxetine-treated group, a significant decrease in the number of green, fluorescent cells was observed, indicating a reduction in ZIKV-infected cells (Figure 5A–C). Quantitative analysis further confirmed statistically significant differences between the ZIKV and dapoxetine groups (Figure 5D), highlighting the effectiveness of dapoxetine in preventing ZIKV infection.

## 3. Discussion

Targeting functional proteins of the ZIKV and searching for specific inhibitors is an important approach in the development of anti-ZIKV drugs. In our study, we identified dapoxetine, an FDA-approved drug that functions as an orally active selective serotonin reuptake inhibitor (SSRI) and is commonly prescribed for the treatment of premature ejaculation (PE). We have observed direct binding of dapoxetine to ZIKV RdRp, resulting in effective inhibition of ZIKV RdRp synthesis. Furthermore, dapoxetine demonstrated EC_50_ values of less than 15 µM against ZIKV in diverse cell models and was able to inhibit the protein expression of ZIKV NS5 and ZIKV E in infected cells, thereby confirming its efficacy against ZIKV infection.

RdRp, MTase, NS2B, and NS3 are important functional proteins in ZIKV, have been the focus of research in the development of targeted therapies [26]. RdRp is involved in the replication and transcription of ZIKV RNA, and drugs such as sofosbuvir, galidesivir, and emetine target this protein [27,28,29]. MTase is responsible for the methylation process of viral RNA, and Sinafenacin, an analog of S-adenosylmethionine (SAM), competitively binds to MTase, inhibiting viral replication [30]. NS3 possesses protease, helicase, and phosphatase activities, the NS2B-NS3 protein complex affects the cleavage of ZIKV polyprotein, while neomycin, bromocriptine and tilmoparfin target the NS2B-NS3 complex [31,32]. Additionally, chloroquine can inhibit ZIKV infection by interfering with viral endocytosis at an early stage [33]. The research and development of new drugs is a complex and long process of exploring new drug discovery pathways and technologies, and although researchers have made significant progress in developing anti-ZIKV drugs, there are currently no approved drugs available on the market. Drug repositioning, also referred to as novel therapeutic applications through drug repurposing, involves discovering new therapeutic uses for drugs that have already been approved for clinical use or are under preclinical or clinical investigation. The repositioning of drugs represents a promising approach for the discovery of novel therapeutic uses [34,35]. This strategy can repurpose existing drugs to treat different diseases or explore new indications by screening marketed drugs against newly discovered disease-causing factors; therefore, the systematic screening of FDA-approved drugs is a promising alternative approach used to accelerate drug development against ZIKV infection [22,23,25]. The emergence of COVID-19 has necessitated novel approaches to drug treatment and presented unprecedented challenges to drug development, thereby highlighting the paramount significance of drug repositioning [36,37]. Numerous studies have demonstrated the potential efficacy of several existing drugs against the SARS-CoV-2 virus [38]. For example, the administration of remdesivir contributed to the rapid recovery of the first confirmed COVID-19 patient in the United States, it has also made remdesivir one of the most considered drugs that may potentially be effective against COVID-19 [39]. This shows that for novel diseases, the discovery of new indications from marketed drugs displays favorable conditions and abundant opportunities. Consequently, an in-depth investigation of the mechanisms of drug action and pathological processes serves as both the foundation and effective approach towards repurposing existing medications. Moreover, this process represents a continuous advancement in our comprehension of disease pathology and drug mechanisms, with the ultimate identification of more efficacious indications within currently available drugs to achieve enhanced therapeutic outcomes. 

Dapoxetine, an orally administered selective serotonin reuptake inhibitor (SSRI), approved by the FDA for treating premature ejaculation (PE), exerts its pharmacological effects by binding to serotonin, norepinephrine, and dopamine reuptake transporters, thereby inhibiting their uptake. Dapoxetine exhibits rapid absorption kinetics, a fast onset of action, and a short half-life, rendering it suitable for on-demand dosing. Clinical trials and applications have demonstrated that dapoxetine exhibits a superior safety and tolerability profile compared to conventional SSRIs, with mild occurrence of common adverse effects such as nausea, diarrhea, headache, and dizziness. Incidences of serious adverse events are infrequent [40,41]. The primary challenge in the search for effective anti-ZIKV drugs lies in their suitability for use in pregnant women and infants, necessitating higher standards for both safety and efficacy [42]. Notably, in the previously mentioned preclinical pharmacology safety studies, in anesthetized Guinea pigs administered with dapoxetine intravenously, the plasma concentrations of dapoxetine ranged from 3902 to 6896 ng/mL (median of 5450 ng/mL), which is approximately 10-fold the clinical C_max_ [43] and higher than the values of anti-ZIKV EC_50_ identified in our study. The above findings suggest that dapoxetine may exhibit a promising safety profile for its potential use as an anti-ZIKV agent in clinical settings, rendering it suitable for administration to pregnant women and infants.

In conclusion, our study identified dapoxetine through computerized docking computational screening of an FDA-approved drug library, further studies showed that dapoxetine has a strong affinity for ZIKV RdRp and can effectively inhibit its RNA synthesis, which has potential for it being repurposed as a ZIKV RdRp inhibitor. Meanwhile, in vivo animal experiments are imperative to investigate dapoxetine’s anti-ZIKV effect, and further studies are needed to confirm the antiviral mechanism of dapoxetine and to explore its potential as a broad-spectrum antiviral drug against other RNA viruses and emerging flaviviruses. Our study has identified a selective serotonin reuptake inhibitor exhibiting antiviral activity and targeting the ZIKV RdRp, thereby offering valuable insights for the development of anti-ZIKV therapeutics. 

## 4. Materials and Methods

### 4.1. Cell Culture, Virus, and Compounds

The material mentioned in this section is reported in our previously published articles [23,24]. The Cell Bank of the Chinese Academy of Sciences (CBCAS), Shanghai, China, is the source of the human lung cancer cell line A549, the African green monkey kidney epithelial cell line Vero, and the human astrocytoma cell line SNB19. These cell lines were cultured in DMEM (Invitrogen, Carlsbad, CA, USA) supplemented with 10% fetal bovine serum (FBS) (GIBCO, Carlsbad, CA, USA), 2 mM L-glutamine, 100 µg/mL streptomycin, and 100 units/mL penicillin (Invitrogen, Carlsbad, CA, USA). The cells were kept in a humidified environment with 5% CO_2_ at 37 °C. At the Medicine Laboratory of the Forensic Medicine Department of Sun Yat-sen University (SYSU) (Guangzhou, China), the cell lines were authenticated using short tandem repeat (STR) fingerprinting, and it was determined that there was no mycoplasma contamination in these cell lines. The ZIKV ZG-01 strain (China, 2016, GenBank accession number KY379148), dapoxetine (catalog no.: HY-B0304A, batch no.: 15163), ribavirin (catalog no.: HY-B0434, batch no.: 11970), and fidaxomicin (catalog no.: HY-17580, batch no.: 15276) were provided in powder form by MedChemExpress LLC (Shanghai, China), along with accompanying quality control document.

### 4.2. Molecular Docking of ZIKV Proteins

For this part of the operation we refer readers to the previous report [22]. The software Molecular Operating Environment (MOE, 2010.10, Chemical Computing Group Inc., Montreal, QC, Canada) was used for molecular docking, using the default settings. The protein structure of the ZIKV NS5 (PDB: 5TFR) was utilized, and the docking site was defined using the MOE Site Finder functionality. The docking results were generated employing the Triangle Matcher method and ASE. The ideal geometric conformation was chosen from among the best results using the Ligand Interactions feature.

### 4.3. Expression and Purification of the ZIKV RdRp protein

Experiments in this section were performed according methods outlined in a previous report [23]. A cDNA fragment of ZIKV RdRp from the ZG-01 strain, containing an N-terminal His-tag sequence, was cloned into a pET-30a-mutant vector. For protein expression, the plasmids were transformed into Rosetta™ competent cells (TIANGEN BIOTECH Co., Ltd., Beijing, China). IPTG was used to induce protein expression, and the cells were harvested for purification using a Ni-NTA affinity column (His-trap HP, GE Healthcare, Beijing, China) in accordance with the recommended protocol from the manufacturer. The concentration of the target protein was determined via SDS-PAGE, with BSA (Sigma, St. Louis, MO, USA) used as a standard.

### 4.4. BIAcore Analysis

BIAcore analysis were performed according to a previous report [22]. Using CM5 sensor chips (General Electric Company, Boston, MS, USA) and a BIAcore T100 device (BIAcore Inc., Uppsala, Sweden), surface plasmon resonance (SPR) experiments were carried out in accordance with the manufacturer’s instructions. Recombinant ZIKV RdRp protein was immobilized on CM5 chips. For three minutes, injections of ribavirin or dapoxetine at varying concentrations were made at a flow rate of 30 µL/min. Subsequently, information was collected for two phases: a 3 min association phase followed and a 20 min dissociation phase. Chip regeneration was achieved by injecting 10 µL of 15 mM NaOH for 20 s. The running buffer for all procedures was 1% DMSO PBS-P20 (GE Healthcare, Beijing, China). Using a 1:1 Langmuir binding model, the binding kinetics were examined using BIA-evaluation software version 3.1. The K_d_ value was calculated following established methods.

### 4.5. ZIKV NS5 RdRp Activity Assay

The Gluc reporter and ZIKV NS5 RdRp plasmids were generously provided by professor Shan Cen from the Institute of Medicinal Biotechnology, Chinese Academy of Medical Sciences, and Peking Union Medical College, Beijing, China. The assay method was as previously described [24,25,44]. HEK-293T cells were co-transfected in a 12-well plate at a density of 1 × 10^5^ cells/mL per well with 5.3 ng of the reporter plasmid and 0.52 μg of the ZIKV RdRp protein expression plasmid. Following transfection, different concentrations of dapoxetine were added. A potent ZIKV NS5 inhibitor, fidaxomicin, at a concentration of 20 μM, was used as a positive control. After 24 h post-transfection, Gluc activity was measured using the Secrete-Pair Dual Luminescence Assay Kit (GeneCopoeia, Rockville, MD, USA). Luminescence readings were acquired using the SpectraMax L Microplate Reader (Molecular Devices, Silicon Valley, San Jose, CA, USA).

### 4.6. Plaque Forming Assay

Plaque assays were performed in accordance with established protocols [22,45]. To ascertain viral titers, Vero cells were seeded in a 12-well plate and incubated at 37 °C with 5% CO_2_ overnight. Subsequently, the medium was removed, and the cells were inoculated with 5-fold serial dilutions of the virus. After a 2 h incubation period, the supernatant was aspirated, and the cells were overlaid with a solution of 2% methyl cellulose in MEM and incubated for 7 days at 37 °C. After being incubated, the cells were cleaned, fixed for 30 min with 4% paraformaldehyde, and stained for 30 min with 2% crystal violet. The viral titers were then expressed as plaque-forming units per milliliter (PFU/mL) after the number of plaques had been counted. DMSO was used as a solvent control.

### 4.7. Cell Viability Assay

MTT experiments were performed with reference to earlier published article [46]. The 3-(4,5-dimethyl-2-thiazolyl)-2,5-diphenyl-2H-tetrazolium bromide (MTT) assay was used to measure the viability of the cells. Briefly, cells were seeded in plates at a density of 8 × 10^3^ cells per well and incubated at 37 °C for 24 h. The cells were exposed to varying dapoxetine concentrations, with DMSO serving as the solvent control. After 48 h, a 5 mg/mL MTT solution was added to the cells and incubated for an additional 4 h before removal. The insoluble formazan was solubilized with 160 mL of DMSO. The absorbance of the 96-well plates was measured at 490 nm using a standard enzyme instrument. The assay was performed in triplicate and repeated three times independently.

### 4.8. RNA Extraction and Real-Time PCR 

This part of the experiment and related reagents are mentioned in previously published article [24]. RIzol reagent (Invitrogen, Carlsbad, CA, USA) was used to isolate RNA from cell supernatant, in accordance with the manufacturer’s instructions. To quantify ZIKV RNA as copies, an internal standard curve consisting of a 7-point dilution series was utilized, employing the FastStart Universal SYBR Green Master Mix (Roche, Basel, Switzerland) in real-time PCR. At least three replicates of each experiment were carried out in triplicate. The 50% effective concentration (EC_50_) was estimated by plotting values from independent triplicate experiments using GraphPad software. The primers used for real-time PCR were as follows: 

ZIKV-E-F 5′-CCGCTGCCCAACACAAG-3′; 

ZIKV-E-R 3′-CCACTAACGTTCTTTTGCAGACAT-5′;

probe 5′-FAM-AGCCTACCTTGACAAGCAGTCAGACACTCAA-3′.

### 4.9. Western Blotting Analysis

The detailed procedure of this experiment is also comprehensively documented in previous report [47]. Cells were grown in 6-well plates at a density of 1 × 10 ^6^ cells per well. After 24 h of incubation, cells were then infected with ZIKV at an MOI of 0.2, and cells were treated with dapoxetine at the indicated concentrations into each well, DMSO was used as the solvent control. At 48 hpi, cells were washed once in iced-cold PBS, then cells were lysed in RIPA lysis buffer (Millipore, Bedford, MA, USA) to extract proteins. The proteins were then separated by sodium dodecyl sulfatepolyacrylamide gel electrophoresis (SDS-PAGE), transferred onto a polyvinylidene difluoride (PVDF) membrane, and blocked with a solution of 5% non-fat milk in Tris-buffered saline buffer for 1 h. The membranes were probed with primary antibodies, including anti-ZIKV E (GTX133314, 1:1000, GeneTex Inc., Irvine, CA, USA), anti-ZIKV NS5 (GTX133312, 1:1000, GeneTex Inc., Irvine, CA, USA), 6x His-tag (ab18184, 1:1000. Abcam, Shanghai, China) and anti-GAPDH (60004-1-IG, 1:7000, Proteintech, Rosemont, IL, USA). Subsequently, the membranes were incubated with a horseradish peroxidase-conjugated secondary antibody, and the signals were visualized by an enhanced chemiluminescence commercial kit (Thermo Fisher Scientific, Waltham, MA, USA), as per the manufacturer’s instructions. 

### 4.10. Immunofluorescence

Immunofluorescence experiments were performed based on previous reports [48,49]. Cells were fixed in 4% paraformaldehyde for 30 min, followed by permeabilization with 0.2% Triton X-100 (Sigma Aldrich, St. Louis, MO, USA) and blocking with 10% BSA in PBS for 1 h. Subsequently, the cells were incubated with an anti-ZIKV E antibody (1:800, BioFront Technologies, Tallahassee, FL, USA) at 4 °C for 12 h. This was followed by incubation with a secondary antibody, Alexa 488-conjugated goat anti-mouse IgG (H + L) (1:500, A32723, Lifetechnologies, Carlsbad, CA, USA), for 1 h. For nuclear counterstaining, 4′,6-diamidino-2-phenylindole (DAPI) stain (1:1000, Sangon Biotech, Shanghai, China) was applied for 15 min at room temperature. Immunofluorescent images were captured using an inverted microscope (Carl Zeiss, Oberkochen, Germany).

### 4.11. Statistical Analysis

All experiments were performed at least three times. Statistical analysis was conducted on triplicate experiments using a two-tailed Student’s *t*-test in GraphPad Prism 8.3.0 software. The results were presented as the mean ± standard deviation (SD). The significance levels were denoted as * *p* < 0.05, ** *p* < 0.01, or *** *p* < 0.001.

## Data Availability

The data presented in this study are available on request from the corresponding author.

## References

[1] Kauffman E.B., Kramer L.D. (2017). Zika Virus Mosquito Vectors: Competence, Biology, and Vector Control. J. Infect. Dis..

[2] Weaver S.C., Costa F., Garcia-Blanco M.A., Ko A.I., Ribeiro G.S., Saade G., Shi P.-Y., Vasilakis N. (2016). Zika virus: History, emergence, biology, and prospects for control. Antivir. Res..

[3] Musso D., Bossin H., Mallet H.P., Besnard M., Broult J., Baudouin L., Levi J.E., Sabino E.C., Ghawche F., Lanteri M.C. (2018). Zika virus in French Polynesia 2013–14: Anatomy of a completed outbreak. Lancet Infect. Dis..

[4] Pierson T.C., Diamond M.S. (2018). The emergence of Zika virus and its new clinical syndromes. Nature.

[5] Beckham J.D., Pastula D.M., Massey A., Tyler K.L. (2016). Zika Virus as an Emerging Global Pathogen: Neurological Complications of Zika Virus. JAMA Neurol..

[6] Cao-Lormeau V.-M., Blake A., Mons S., Lastère S., Roche C., Vanhomwegen J., Dub T., Baudouin L., Teissier A., Larre P. (2016). Guillain-Barré Syndrome outbreak associated with Zika virus infection in French Polynesia: A case-control study. Lancet.

[7] Dibo M., Battocchio E.C., dos Santos Souza L.M., da Silva M.D.V., Banin-Hirata B.K., Sapla M.M.M., Marinello P., Rocha S.P.D., Faccin-Galhardi L.C. (2019). Antibody Therapy for the Control of Viral Diseases: An Update. Curr. Pharm. Biotechnol..

[8] Barouch D.H., Thomas S.J., Michael N.L. (2017). Prospects for a Zika Virus Vaccine. Immunity.

[9] White J., Tunga P., Anderson D.M., Iledan K., Loreth T., Parrera G.S., Astacio H., Drobic B., Richardson J.S. (2021). Results of a Double-Blind, Randomized, Placebo-Controlled Phase 1 Study to Evaluate the Safety and Pharmacokinetics of Anti-Zika Virus Immunoglobulin. Am. J. Trop. Med. Hyg..

[10] Baz M., Boivin G. (2019). Antiviral Agents in Development for Zika Virus Infections. Pharmaceuticals.

[11] Boldescu V., Behnam M.A.M., Vasilakis N., Klein C.D. (2017). Broad-spectrum agents for flaviviral infections: Dengue, Zika and beyond. Nat. Rev. Drug Discov..

[12] Kostyuchenko V.A., Lim E.X.Y., Zhang S., Fibriansah G., Ng T.-S., Ooi J.S.G., Shi J., Lok S.-M. (2016). Structure of the thermally stable Zika virus. Nature.

[13] Rey F.A., Stiasny K., Heinz F.X. (2017). Flavivirus structural heterogeneity: Implications for cell entry. Curr. Opin. Virol..

[14] Zhao B., Yi G., Du F., Chuang Y.-C., Vaughan R.C., Sankaran B., Kao C.C., Li P. (2017). Structure and function of the Zika virus full-length NS5 protein. Nat. Commun..

[15] Brecher M., Chen H., Li Z., Banavali N.K., Jones S.A., Zhang J., Kramer L.D., Li H. (2015). Identification and Characterization of Novel Broad-Spectrum Inhibitors of the Flavivirus Methyltransferase. ACS Infect. Dis..

[16] De Clercq E., Li G. (2016). Approved Antiviral Drugs over the Past 50 Years. Clin. Microbiol. Rev..

[17] Lamb Y.N. (2020). Remdesivir: First Approval. Drugs.

[18] Jayk Bernal A., Gomes da Silva M.M., Musungaie D.B., Kovalchuk E., Gonzalez A., Delos Reyes V., Martín-Quirós A., Caraco Y., Williams-Diaz A., Brown M.L. (2022). Molnupiravir for Oral Treatment of Covid-19 in Nonhospitalized Patients. New Engl. J. Med..

[19] Deng G., Li D., Sun Y., Jin L., Zhou Q., Xiao C., Wu Q., Sun H., Dian Y., Zeng F. (2023). Real-world effectiveness of Azvudine versus nirmatrelvir–ritonavir in hospitalized patients with COVID-19: A retrospective cohort study. J. Med. Virol..

[20] Ramharack P., Soliman M.E.S. (2017). Zika virus NS5 protein potential inhibitors: An enhanced in silico approach in drug discovery. J. Biomol. Struct. Dyn..

[21] Duan W., Song H., Wang H., Chai Y., Su C., Qi J., Shi Y., Gao G.F. (2017). The crystal structure of Zika virus NS5 reveals conserved drug targets. EMBO J..

[22] Yuan J., Yu J., Huang Y., He Z., Luo J., Wu Y., Zheng Y., Wu J., Zhu X., Wang H. (2020). Antibiotic fidaxomicin is an RdRp inhibitor as a potential new therapeutic agent against Zika virus. BMC Med..

[23] Zhu Y., Liang M., Yu J., Zhang B., Zhu G., Huang Y., He Z., Yuan J. (2023). Repurposing of Doramectin as a New Anti-Zika Virus Agent. Viruses.

[24] Zhu Y., Yu J., Chen T., Liu W., Huang Y., Li J., Zhang B., Zhu G., He Z., Long Y. (2023). Design, synthesis, and biological evaluation of a series of new anthraquinone derivatives as anti-ZIKV agents. Eur. J. Med. Chem..

[25] Zhou R., Li Q., Yang B., Quan Y., Liu Y., Liu M., Zhang Y., Shan G., Li Z., Wang J. (2022). Repurposing of the antihistamine mebhydrolin napadisylate for treatment of Zika virus infection. Bioorg. Chem..

[26] Bernatchez J.A., Tran L.T., Li J., Luan Y., Siqueira-Neto J.L., Li R. (2019). Drugs for the Treatment of Zika Virus Infection. J. Med. Chem..

[27] Bullard-Feibelman K.M., Govero J., Zhu Z., Salazar V., Veselinovic M., Diamond M.S., Geiss B.J. (2017). The FDA-approved drug sofosbuvir inhibits Zika virus infection. Antivir. Res..

[28] Julander J.G., Demarest J.F., Taylor R., Gowen B.B., Walling D.M., Mathis A., Babu Y.S. (2021). An update on the progress of galidesivir (BCX4430), a broad-spectrum antiviral. Antivir. Res..

[29] Yang S., Xu M., Lee E.M., Gorshkov K., Shiryaev S.A., He S., Sun W., Cheng Y.-S., Hu X., Tharappel A.M. (2018). Emetine inhibits Zika and Ebola virus infections through two molecular mechanisms: Inhibiting viral replication and decreasing viral entry. Cell Discov..

[30] Zhang J., Zheng Y.G. (2015). SAM/SAH Analogs as Versatile Tools for SAM-Dependent Methyltransferases. ACS Chem. Biol..

[31] Nunes D.A.D.F., Santos F.R.D.S., da Fonseca S.T.D., de Lima W.G., Nizer W.S.D.C., Ferreira J.M.S., de Magalhães J.C. (2021). NS2B-NS3 protease inhibitors as promising compounds in the development of antivirals against Zika virus: A systematic review. J. Med. Virol..

[32] Kang C., Keller T.H., Luo D. (2017). Zika Virus Protease: An Antiviral Drug Target. Trends Microbiol..

[33] Shiryaev S.A., Mesci P., Pinto A., Fernandes I., Sheets N., Shresta S., Farhy C., Huang C.-T., Strongin A.Y., Muotri A.R. (2017). Repurposing of the anti-malaria drug chloroquine for Zika Virus treatment and prophylaxis. Sci. Rep..

[34] Corsello S.M., Bittker J.A., Liu Z., Gould J., McCarren P., Hirschman J.E., Johnston S.E., Vrcic A., Wong B., Khan M. (2017). The Drug Repurposing Hub: A next-generation drug library and information resource. Nat. Med..

[35] Sonaye H.V., Sheikh R.Y., Doifode C.A. (2021). Drug repurposing: Iron in the fire for older drugs. Biomed. Pharmacother..

[36] Singh T.U., Parida S., Lingaraju M.C., Kesavan M., Kumar D., Singh R.K. (2020). Drug repurposing approach to fight COVID-19. Pharmacol. Rep..

[37] Ng Y.L., Salim C.K., Chu J.J.H. (2021). Drug repurposing for COVID-19: Approaches, challenges and promising candidates. Pharmacol. Ther..

[38] Zhao M., Zhang J., Li H., Luo Z., Ye J., Xu Y., Wang Z., Ye D., Liu J., Li D. (2021). Recent progress of antiviral therapy for coronavirus disease 2019. Eur. J. Pharmacol..

[39] Holshue M.L., DeBolt C., Lindquist S., Lofy K.H., Wiesman J., Bruce H., Spitters C., Ericson K., Wilkerson S., Tural A. (2020). First Case of 2019 Novel Coronavirus in the United States. N. Engl. J. Med..

[40] Feige A.M., Pinsky M.R., Hellstrom W.J.G. (2010). Dapoxetine for Premature Ejaculation. Clin. Pharmacol. Ther..

[41] Castiglione F., Albersen M., Hedlund P., Gratzke C., Salonia A., Giuliano F. (2016). Current Pharmacological Management of Premature Ejaculation: A Systematic Review and Meta-analysis. Eur. Urol..

[42] Tebas P., Roberts C.C., Muthumani K., Reuschel E.L., Kudchodkar S.B., Zaidi F.I., White S., Khan A.S., Racine T., Choi H. (2021). Safety and Immunogenicity of an Anti–Zika Virus DNA Vaccine. N. Engl. J. Med..

[43] Kowey P.R., Mudumbi R.V., Aquilina J.W., Dibattiste P.M. (2011). Cardiovascular Safety Profile of Dapoxetine during the Premarketing Evaluation. Drugs R&D.

[44] Yao Z.-W., Liu H., Zhou R., Feng M.-Y., Wang F., Qin X.-J., Chen X.-X., Zheng C.-B., Luo R.-H., Yang L.-M. (2021). Non-volatile acylphloroglucinol components from Eucalyptus robusta inhibit Zika virus by impairing RdRp activity of NS5. Bioorganic Chem..

[45] Vicenti I., Boccuto A., Giannini A., Dragoni F., Saladini F., Zazzi M. (2018). Comparative analysis of different cell systems for Zika virus (ZIKV) propagation and evaluation of anti-ZIKV compounds in vitro. Virus Res..

[46] Lin C., Yu J., Hussain M., Zhou Y., Duan A., Pan W., Yuan J., Zhang J. (2018). Design, synthesis, and biological evaluation of novel 7-deazapurine nucleoside derivatives as potential anti-dengue virus agents. Antivir. Res..

[47] He Z., Chen J., Zhu X., An S., Dong X., Yu J., Zhang S., Wu Y., Li G., Zhang Y. (2018). NLRP3 Inflammasome Activation Mediates Zika Virus–Associated Inflammation. J. Infect. Dis..

[48] Leiva S., Dizanzo M.P., Fabbri C., Bugnon Valdano M., Luppo V., Levis S., Cavatorta A.L., Morales M.A., Gardiol D. (2021). Application of quantitative immunofluorescence assays to analyze the expression of cell contact proteins during Zika virus infections. Virus Res..

[49] Zou M., Li J.-Y., Zhang M.-J., Li J.-H., Huang J.-T., You P.-D., Liu S.-W., Zhou C.-Q. (2021). G-quadruplex binder pyridostatin as an effective multi-target ZIKV inhibitor. Int. J. Biol. Macromol..

